# Editorial: Computer-Aided Biodesign Across Scales

**DOI:** 10.3389/fbioe.2021.700418

**Published:** 2021-06-15

**Authors:** Thomas E. Gorochowski, Jonathan R. Karr, Fabio Parmeggiani, Boyan Yordanov

**Affiliations:** ^1^School of Biological Sciences, University of Bristol, Bristol, United Kingdom; ^2^BrisSynBio, University of Bristol, Bristol, United Kingdom; ^3^Department of Genetics and Genomic Sciences, Icahn School of Medicine at Mount Sinai, New York, NY, United States; ^4^School of Chemistry and School of Biochemistry, University of Bristol, Bristol, United Kingdom; ^5^Scientific Technologies Ltd., London, United Kingdom; ^6^Microsoft Research, Cambridge, United Kingdom

**Keywords:** biodesign, synthetic biology, bioengineering, multi-scale, systems biology, computational modeling, protein design, biocomputation

## Introduction

**Figure 1:**
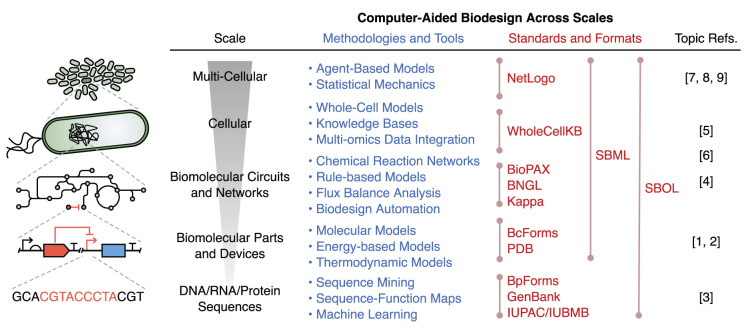
Overview of computer-aided biodesign methodologies, tools, and standards used across scales. Although each methodology/tool has been placed at the specific scale it is commonly used, it should be noted that many can be applied across scales. Lines in the “Standards and Formats” section denote the range of scales over which the standard/format can be used. BioPAX, Biological Pathway Exchange; BNGL, BioNetGen Language; SBML, Systems Biology Markup Language; SBOL, Synthetic Biology Open Language; PDB, Protein Data Bank.

Computer-aided design (CAD) has revolutionized many engineering fields, enabling the quick exploration and testing of designs *in silico*, that minimizes the need for expensive and laborious physical assembly and experimentation. We believe that CAD will become similarly important to synthetic biology. However, synthetic biology presents some unique challenges for CAD, including the multi-scale structure of biology, the combinatorial complexity of molecular systems due the low degree of insulation inside cells, the stochastic nature of many biological processes, and our limited ability to accurately characterize the components of these living systems. Despite these challenges, numerous advancements are being made toward CAD for many aspects of biodesign. These advances are accelerating our abilities to efficiently assemble synthetic biological systems and revealing underlying principles for their effective design. In this Research Topic we have collated a broad range of original research, perspectives, and reviews covering some of the current approaches to computer-aided biodesign across scales (Figure 1).

## *De Novo* Molecular Prediction and Design

At the molecular level, the design of proteins and DNA sequences pose many challenges, but reliable modeling at this level will offer the means to scale-up biodesign, moving our focus from single molecules to complex multi-component systems. To make this step, modeling and design tools need to be able to recapitulate experimental data.

To address this need, Frenz et al. analyze the ProTherm database, which contains thermodynamic information about large numbers of protein mutations, to build a broader understanding of potential biases and to develop a curated subset to improve the prediction of mutational effects. Such robust inference is at the core of traditional protein design methods and Yeh et al. push these approaches further by developing an interactive user interface (Elfin UI) to build proteins and protein complexes with arbitrary shapes from compatible structural building blocks. These architectures are much larger (in the range of thousands of amino acids) than routinely designed proteins and begin to cross the boundary from the molecular to the cellular scale. Modeling tools are also central to the prediction of desirable features at a genetic level. Gou et al. describe SSRMMD, an algorithm for identification of microsatellites (or simple sequence repeats, SSRs) within genomes to allow for better navigation and comparison of genomes.

## Toward the Design of Cell-Free Systems and Whole Cells

Due to the complexity of molecular biology, the molecular to cell scale is one of the most difficult scales of biology to design. Designing cells requires computational systems that help engineers navigate numerous challenges, including the large number of distinct molecular species involved in extant cells and the lack of rigid structures or insulation between parts, which gives rise to an extraordinary number of molecular interactions.

One potential way to circumvent these challenges is to focus on cell-free systems, which promise to be easier to construct, control, test, and model. Laohakunakorn highlights these advantages and describes how the reduced complexity of cell-free systems could be a powerful training ground for model-driven design of cells. Due to the limitations of cell-free systems, many problems will likely require engineering complete synthetic cells. For example, cells would likely be easier to deploy in open environments than cell-free systems. Designing entire cells requires confronting the complexity of molecular biology. Marucci et al. describe the models that will be needed to tackle this challenge and how such models could revolutionize synthetic biology. Achieving such models will likely require the collaborative efforts of numerous modelers, experimentalists, and engineers, which in turn will likely require standards for exchanging information about synthetic biological systems. Foreseeing this need, McLaughlin et al. report a new version of the Synthetic Biology Open Language (SBOL) which is substantially easier to use. McLaughlin et al. anticipate that the new version of SBOL will accelerate the adoption of SBOL and, in turn, the collaborative development of more sophisticated synthetic biological systems.

## Computer-Aided Biodesign Beyond Single Cells

Most synthetic biology efforts to date have focused on the design of individual cells with basic functionalities (e.g., implementing basic logic). However, outside the lab cells rarely exist in isolation and their ability to interact through chemical signaling and the inherent heterogeneity in cellular states across a population due to environmental perturbations can act as a basis for important collective behaviors. These emergent properties need to be understood even for simple synthetic circuits to function reliably and can even be exploited to create more robust or scalable distributed biological computations. However, designing individual cells to exhibit desired population-level behaviors is challenging, requiring novel computational and theoretical approaches.

Gorochowski et al. propose that multi-agent modeling could serve as a design framework for engineering living collectives and offer a way to better understand the underlying causes and driving factors of emergent properties in protocellular systems, developmental programs, disease states and industrial bioprocesses. Karkaria et al. present additional examples where the engineering of monocultures in synthetic biology has reached a bottleneck. Distributed computing is reviewed as a theoretical framework for understanding and designing distributed multicellular systems in biology to overcome these limitations. They consider a wide range of distributed algorithms used by biology covering the use of bet hedging, organism development and bacterial colony formation. Finally, Feliú et al. present original research spanning biological scales to understand and tune the patterns that emerge within a population of cells where each cell contains an identical synthetic oscillator circuit. They use computational modeling spanning multiple scales and show how a simplified cellular model coupled to varying environmental conditions can provide a convenient design tool that closely matches more complex multi-agent simulations.

## Conclusion

Being able to scale our ability to harness biology will be crucial for addressing the many grand challenges we face, such as shifts toward sustainable manufacturing, clean energy production, and new forms of advanced medicine. CAD applied to synthetic biology is likely to play a key role in realizing these ambitions and the articles presented in this topic provide a broad introduction to CADs current role, in addition to a glimpse at its possible development and integration into the bioengineering practices of the future.

## Author Contributions

All authors listed have made a substantial, direct and intellectual contribution to the work, and approved it for publication.

## Conflict of Interest

BY was employed by the companies Scientific Technologies Ltd. and Microsoft Research. The remaining authors declare that the research was conducted in the absence of any commercial or financial relationships that could be construed as a potential conflict of interest.

